# Assessment of dose distribution in pancreatic SABR in the presence of a metallic biliary stent using Monte Carlo modeling

**DOI:** 10.1002/acm2.70712

**Published:** 2026-07-22

**Authors:** Ahmad Abbas, Owen Nicholas, Richard P. Hugtenburg

**Affiliations:** ^1^ Swansea University Medical School Swansea University Swansea Wales UK; ^2^ Adan Hospital Ministry of Health Hadiya Kuwait; ^3^ South West Wales Cancer Centre Swansea Bay University Health Board Swansea Wales UK

**Keywords:** biliary stent, dose distribution, metal artifact, Monte Carlo simulation, nitinol, pancreatic cancer, PRIMO, stereotactic ablative radiotherapy (SABR), VMAT

## Abstract

**Background:**

Stereotactic ablative radiotherapy (SABR) with dose escalation is increasingly used for pancreatic cancer; however, the dosimetric impact of metallic biliary stents remains poorly characterized. Self‐expanding metal stents (SEMS), commonly placed for obstructive jaundice, contain high‐density nitinol that produces computed tomography (CT) artifacts, including beam hardening, potentially compromising treatment planning system (TPS) dose accuracy. Precise dose estimation is critical given the proximity of the duodenum and other organs at risk (OARs).

**Purpose:**

This study evaluated dose distribution in pancreatic SABR in the presence of a metallic biliary stent using Monte Carlo (MC) simulation with refined stent contouring and material correction.

**Methods:**

Two patients with SEMS (Cook Evolution, nitinol alloy) treated with SABR (50 Gy in 5 fractions) using volumetric modulated arc therapy (VMAT) were retrospectively analyzed. Plans were generated in Eclipse TPS (v.13.7) using the analytical anisotropic algorithm. MC simulations were performed in PRIMO (PENELOPE‐based) with ≤2% uncertainty. A novel contouring technique separated the hollow lumen from the metallic stent wall to correct CT beam hardening artifacts. Nitinol (density 6.45 g/cm^3^) was incorporated into MC calibration. Comparisons between standard MC, modified MC, and TPS were assessed using percentage agreement (PA), gamma passing rate (GPR; 3%/3 mm), and dose‐volume histogram metrics.

**Results:**

Refined contouring reduced modeled stent volumes from 26.1 to 22.8 cm^3^ and from 19.87 to 18.23 cm^3^. The standard MC model overestimated planning target volume (PTV) dose by 14.9% and 15.7%, with PTV GPRs of 55.29% and 79.78%, respectively. Approximately 2% dose enhancement was observed at the stent‐tissue interface. Compared with the modified MC model, TPS underestimated dose lateral to the stent by up to 12.3% (worst case, Patient B, Plan 2) and overestimated it elsewhere by up to 12.2%. The duodenum showed the largest OAR discrepancies, with TPS up to 9.7% underestimation adjacent to the stent, whereas distant structures demonstrated high agreement.

**Conclusion:**

Conventional TPS algorithms exhibit clinically relevant inaccuracies in the presence of metallic biliary stents. Incorporating refined stent contouring and material correction within an MC framework improves dosimetric accuracy and may support safer dose escalation in pancreatic SABR.

## 1 INTRODUCTION

Pancreatic cancer is the 10^th^ most common cancer in the UK, accounting for over 10,000 new cases annually. It remains one of the most lethal malignancies, with 10‐year survival rates of approximately 5%.^1^ For early stage tumors, the cornerstone of treatment is surgery, often with chemotherapy administered in the adjuvant setting. Radiotherapy is commonly used, particularly in patients with locally advanced or borderline resectable tumors. However, the role of radiotherapy in the treatment paradigm for pancreatic cancer remains uncertain because of the ambiguous results of trials evaluating its benefit.^2^, ^3^, ^4^, ^5^ An inadequate dose has been proposed as a potential reason for the limited effectiveness of radiotherapy in pancreatic cancer, as reflected by poor local control rates. Autopsy data indicate that more than 30% of patients die from local disease progression.^6^ Reported response rates for doses < 60 Gy range between 5‐% and 25%.^7^ A recent study evaluating dose escalation to maximize dose‐response concluded that a dose of 80 Gy (biological equivalent dose [BED] 10) may achieve response rates of up to 50%.^7^ Other studies have suggested improved patient survival when 70 Gy (BED) was delivered.^8^, ^9^ These findings have led to the development of stereotactic ablative radiotherapy (SABR) for pancreatic cancer. SABR offers several advantages over conventional radiotherapy. One key benefit is increased dose conformality, which enhances normal tissue sparing and allows dose escalation to achieve a higher BED in radioresistant tumors.^10^, ^11^ Several studies have demonstrated the safety and efficacy of regimens up to 50 Gy/5, corresponding to a BED10 > 100 Gy.^12^, ^13^, ^14^ However, dose escalation may lead to unacceptable toxicity, particularly in the duodenum, which is often in close proximity to the pancreas. Significant toxicities include ulcers, bleeding, strictures, and bowel perforation.^15^ Other nearby organs at risk (OARs) include the stomach, small and large intestines, kidneys, and liver. An additional obstacle to dose escalation is intrafractional pancreatic movement during respiration and digestion.

To mitigate these risks, magnetic resonance‐guided linear accelerator (MR‐Linacs) have been implemented to deliver superior imaging coupled with an online adaptive workflow.^12^, ^16^ This technology improves precision and clinician confidence in treatment delivery.^17^, ^18^ however, it is resource‐intensive and therefore not widely available.^19^, ^20^, ^21^

Pancreatic cancer commonly presents with painless jaundice due to biliary obstruction secondary to a periampullary tumor. Initial management of obstructive jaundice typically involves biliary drainage via endoscopic placement of a biliary stent.^22^ Biliary stents vary in shape and material including silicon‐covered stents, single bare‐metal stents, double bare‐metal stents, and large open‐cell stents.^22^ Self‐expanding metal stents (SEMS) are commonly used and are typically composed of a nickel‐titanium alloy (nitinol) mesh.^23^ The delivery of external beam radiotherapy in the presence of a metal stent may lead to dose perturbations due to secondary electron production and scatter.^8^ Few studies have examined the effect of biliary stents on dose distribution in pancreatic radiotherapy. With the emergence of dose‐escalation approaches, interest has increased in understanding high‐dose effects on both the target and surrounding OARs.^24^, ^25^, ^26^ Lee et al. investigated the impact of different stent designs on dose distribution using film dosimetry in a phantom model. A single beam was used to irradiate a phantom containing a stent. Dose enhancement around the stent ranged from 2.3‐% to 8.2% due to scattered radiation, with the highest enhancement (19.5 %) observed for the double bare stent. Conversely, dose reductions of between 0.9% and 3.6% were observed distal to the stent. The use of multiple beams or volumetric modulated arc therapy (VMAT) reduced scatter‐related effects. Four‐beam arrangements resulted in dose enhancements of 6.7%‐14.9% depending on stent design, whereas VMAT showed a mean enhancement of 4.8% and a maximum of 15.3% for the double bare‐metal stent. VMAT also reduced cold and hot spots caused by beam overlap from different directions. However, high calculation accuracy remains essential to ensure that OARs near the stent, including the kidneys, liver, small bowel, stomach, duodenum, and spinal cord, do not exceed dose tolerance thresholds. A comparison between radiopaque marker measurements and treatment planning system (TPS) calculations showed a 12.8% underestimation of proximal dose in single‐beam simulations, while distal doses were overestimated by 9.6%.^26^

The present study aimed to evaluate dose distribution using the gold‐standard Monte Carlo (MC) algorithm in patients with a biliary stent. The stent material composition was modified to match its actual components in order to improve MC simulation accuracy and correct for computed tomography (CT) artifacts. Additionally, a novel contouring technique was used to more accurately represent the stent anatomy within the biliary duct.

## 2 METHODS

Two anonymized clinical cases from our institutional database were included in this study. Both patients (Patient A and Patient B) underwent SEMS insertion as part of the initial management of obstructive jaundice prior to radiotherapy. A Cook biliary stent was used at our institution.^27^ The stent is composed of nitinol, a nickel‐titanium alloy. Target volumes and OARs were delineated by a consultant clinical oncologist. The anonymized Digital Imaging and Communications in Medicine (DICOM) datasets were imported into the TPS Eclipse v.13.7 (Varian Medical Systems, Palo Alto, CA). Both patients were treated on a Varian TrueBeam linear accelerator with 6 MV photons using single full‐arc VMAT technique (Millennium 120‐leaf MLC). Plan were generator using analytical anisotropic algorithm (AAA). Structure delineation was performed in Mirada Medical (v.1.8.6). For each patient, two VMAT plans were created with a prescribed dose of 50 Gy in five fractions. The first plan prioritized planning target volume (PTV) coverage (Plan 1), whereas the second prioritized OAR constraints (Plan 2). The planning constraints were those routinely used in our institution and are summarized in Table 1. The DICOM datasets, including radiotherapy plans, were then prepared for MC simulation. PRIMO was used as the MC simulation platform.^28^ The two patients analyzed constitute the complete eligible cohort identified in our institutional database during the study window, that is, all patients with self‐expanding metal biliary stent in situ at CT simulation who subsequently received pancreatic SABR (50 Gy in 5 fraction) and whose DICOM‐RT datasets were of sufficient quality for retrospective Monte Carlo recalculation. The same contouring/material correction methodology was previously applied to these patients under conventional fractionation (50.4 Gy in 28 fractions) using both single‐beam and VMAT geometries, yielding consistent dose‐perturbation patterns across all beam configurations and fractionation protocols. The present technical note therefore demonstrates and characterizes the methodology under SABR conditions; prospective multi‐patient evaluation and phantom‐based experimental verification are identified as essential next steps. The requirement for informed consent was waived due to the retrospective nature of the study, which involved analysis of existing data with no direct patient contact and minimal risk to participants. All data were anonymized prior to analysis.

**TABLE 1:** SABR constraints used for VMAT plan creation in five fractions.^29^

Structure	Metric	Optimal constraint	Mandatory constraint
Duodenum	D_0.5 cc_	33 Gy	35 Gy
	D_10 cc_	25 Gy	‐
Spinal canal	D_0.035 cc_	‐	25.3 Gy
Stomach	D_0.5 cc_	33 Gy	35 Gy

SABR: stereotactic ablative radiotherapy; VMAT: volumetric modulated arc therapy; D0.5 cc, D10 cc, and D0.035 cc: minimum dose received by 0.5 cm^3^, 10 cm^3^, and 0.035 cm^3^ of the specified structure, respectively.

### 2.1 Primo MC preparation

Monte Carlo simulations were performed using PRIMO version 0.3.65.1814, 64‐bit,^28^ based on PENELOPE general‐purpose Monte Carlo radiation‐transport code.^30^ The nitinol material file was generated using pyPENELOPE 2011 with a 50%/50% nickel‐titanium weight fraction at a physical density of 6.45 g/cm^3^.

The PRIMO Monte Carlo dose engine was commissioned by tunning the initial‐electron beam parameters to reproduce measured commissioning data (depth‐dose curves and lateral profile for a 10×10 cm^2^ 6 MV field at SSD 100 cm in water phantom). Following commissioning, agreement between PRIMO and the TPS (Eclipse AAA) in homogenous water was verified, with a global gamma (3%/3 mm) passing rate of 99%. This agreement in homogenous geometry does not constitute experimental validations in heterogeneous geometric containing high‐Z material, that limitation is addressed in section 4.

Within the PRIMO environment, the stent‐wall Hounsfield units were override to 3500 HU (mapping to nitinol via the modified CT to material calibration curve, on the PRIMO scale where 0 HU = air), and the inner‐lumen HU was set to 1000 HU (water‐equivalent) to correct the beam‐hardening cupping artefact (which had reduced the CT measured inner‐lumen HU to approximately ‐500). A new geometric calibration incorporating nitinol was then applied.

The CT derived voxelised geometry used a dose grid of 2.5 × 2.5 × 3.0 mm^3^ (160 × 120 × 133 voxels), matched to the TPS calculated grid. A splitting ratio of 1000 was applied as the primary variance reduction technique. The phase space file was generated with sufficient histories to achieve a statistical uncertainty of ≤2% (2σ) at the prescription isodose, with 4.6 × 10^10^ histories simulated. Phase space generation required approximately 340 hours, and each VMAT simulation approximately 44 hours, on an Intel i7‐9700 processor (3.0 GHz, 12 MB cache).

For each patient, three simulations were conducted. The first simulation was performed without HU modification and is referred to as the standard MC model. The second simulation included modification of the stent material using the new contouring technique and is called the modified MC plan (Plan 1). The third simulation used the modified model for plan 2. MC results were compared with TPS calculations using percentage agreement (PA) and gamma analysis with criteria ∆D = 3% and ∆d = 3 mm, reporting the gamma passing rate (GPR).^31^ The AAPM TG‐218 report recommends 3%/2 mm with a 95% universal tolerance as the contemporary IMRT/VMAT QA standard, with tighter criteria appropriate for SABR; the implications of criterion choice for the present findings are addressed in section 4.^32^

### 2.2 New contouring model for the stent

A new contouring approach was implemented to improve representation of the stent geometry. The contouring workflow produced three DICOM RT structures for each patient:

- Stent, the original full stent contour surrounding both metallic mesh and the inner lumen, delineated in the planning CT.
- Inside stent, the inner lumen contour, delineated as the contiguous low ‐HU core (‐500 HU on the planning CT, attributable to beam hardening cupping artefact, and clearly distinguishable from bile at +10 to +30 HU and from the saturated high HU metallic mesh). The boundary was identified slice by slice using narrow window/level setting tuned to maximize mesh‐lumen contrast and refined manually where partial‐volume averaging blurred the transition.
- New model stent, the corrected stent contour. Representing only the metallic mesh for nitinol material assignment.

**FIGURE 1:**
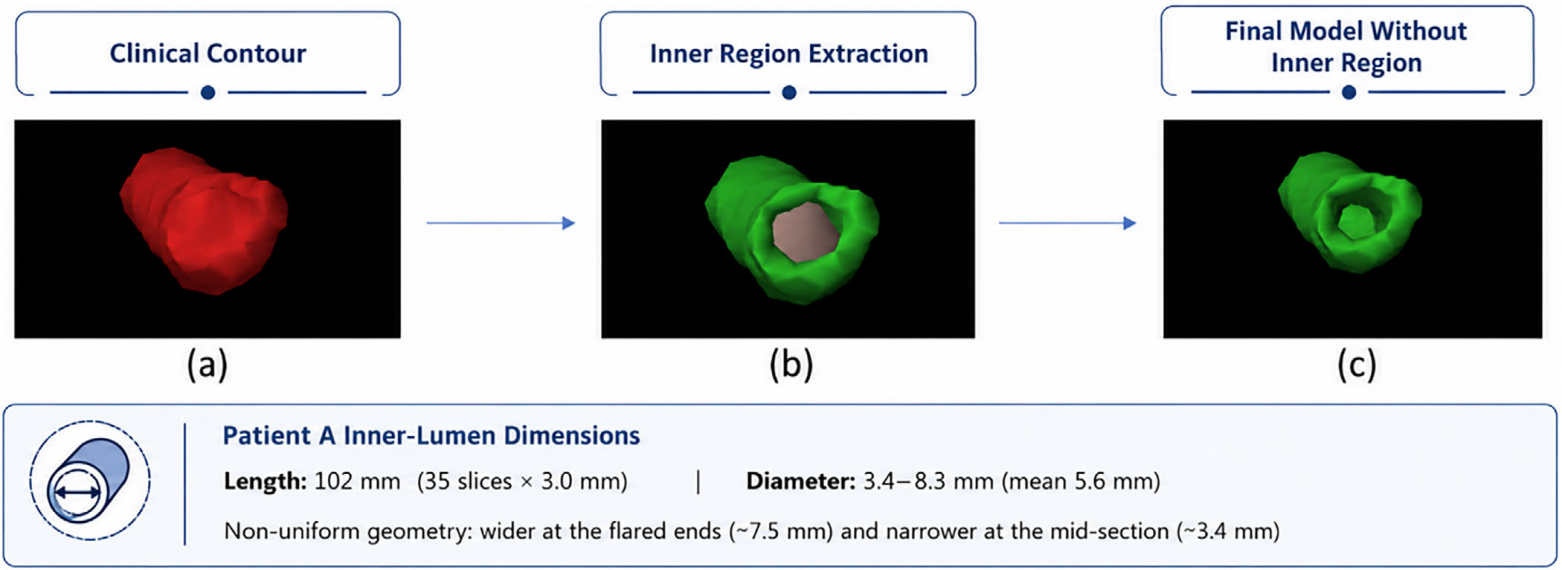
The new contouring model extracts the inner region of the stent. (a) Original contouring in which the entire region was included. (b) The inner region was contoured, and separation can be visualized. (c) The inner region is extracted, and only the stent is included.

The inner contour was non uniform along the stent axis because (a) the deployed STEMS exhibits flared end geometry, (b) physiological compression by adjacent tissue causes minor in plane lumen deformation, and (c) beam hardening severity varies with the angular distribution of CT projections through the metallic mesh. The contouring/material correction approach was chosen over direct geometric modelling of the stent mesh for example STL mesh for reasons of voxels resolution compatibility, clinical translatability, and design uncertainty, as discussed in section 4. For patient A, the inner‐lumen length was 102 mm with effective inner diameter ranging from 3.4 to 8.3 mm (mean 5.6 mm), narrowest at the mid‐section and widest at the flared ends. For patient B, the inner‐lumen length was 60 mm with effective inner diameter ranging from 2.8 mm to 8.6 mm (mean 5.8 mm) (Figure 1).

## 3 RESULTS

### 3.1 Comparison between modified MC model and standard MC model

The PRIMO MC simulations achieved a statistical uncertainty of ≤ 2%. For Patient A, the modified model reduced the stent volume from 26.1 cm^3^ to 22.8 cm^3^. The standard MC model overestimated the PTV dose by 14.9% compared with the modified model. A 9% difference was observed for the duodenum. The stomach, spleen, and spinal cord demonstrated minimal differences between models (≤1.1%). A localized dose enhancement of approximately 2.1% was observed in the modified MC model at the proximal stent edge (for example, at the entry side of the metallic mesh in the dominant photon path). This feature was absent or substantially smeared in the standard MC model, in which the entire stent region was modelled as a uniform high‐density volume and incorrected CT HU assignment treated the stent interior as bone equivalent material. Detailed results are summarized in Table 2.

**TABLE 2:** Comparison of the modified MC model and the standard MC model for Patients A and B.

					Difference		
Modified MC model vs Standard MC model	Std MC mean (Gy)	Mod MC Mean (Gy)	Percentage of agreement (PA) %	Gamma passing rate (GPR) %	D95%	D50%	D5%
**Patient A**							
SABR_PTV	49.5	47.2	88.06	55.29	14.27	9.6	4.58
Duodenum	21.5	20.1	93.28	97.07	0.2	0.93	8.43
Stomach	2.7	2.7	92.5	100	0	0.23	1.4
Spinal cord	3.0	3.0	95.8	100	0	0	0.96
Spleen	2.0	2.0	91	100	0	0.39	1
**Patient B**							
SABR_PTV	55.8	56	94.9	79.78	14.11	3	0.16
Duodenum	37.5	36.9	94.08	96.69	2.51	3.67	2.47
Stomach	12.6	12.6	98.66	99.98	0	0.13	1.1
Spinal cord	10.0	10.0	97.35	100	0	0.32	0.75
Spleen	5.1	5.1	98.78	100	0	0	0.47

MC: Monte Carlo; PA: percentage agreement; GPR: gamma passing rate (3%/3 mm criteria); PTV: planning target volume; SABR_PTV: stereotactic ablative radiotherapy planning target volume; D95%, D50%, and D5%: dose received by 95%, 50%, and 5% of the structure volume, respectively. Std MC = standard MC model (without HU correction). Mod MC = modified MC model (with corrected stent material).

For Patient B, the stent volume decreased from 19.87 cm^3^ to 18.23 cm^3^ using the modified model. The standard MC model overestimated the PTV dose by 15.7%, with a reduced GPR. The duodenum showed a 4.61% difference between models, again with overestimation near the stent‐PTV interface. A dose enhancement of approximately 2% was observed at the duodenal border adjacent to the stent.

### 3.2 Comparison between modified MC model and TPS

For Patient A (Plan 1), TPS underestimated the PTV dose by up to 8% lateral to the stent and near the duodenum, while overestimation reached 12.2% in other regions. The GPR was 67.87% with 94.8% PTV agreement (Table 3). The dose‐volume histogram (DVH) showed a higher mean PTV dose in TPS (54 Gy) compared with 47.2 Gy in the MC simulation.

**TABLE 3:** Results of Plan 1 (PTV‐prioritized) and Plan 2 (OAR‐prioritized): Comparison between the modified MC model and TPS for Patient A and B.

					Difference		
Modified MC model vs TPS	MC Mean (Gy)	TPS Mean (Gy)	Percentage of agreement (PA) %	Gamma passing rate (GPR) %	D95%	D50%	D5%
**Patient A Plan 1**					
SABR_PTV	47.2	52.4	94.8	67.87	10.72	6.15	0.24
Duodenum	20.1	21.4	93	96	0.68	0.5	4.6
Stomach	2.7	2.7	95.74	100	0.19	0	0
Spinal cord	3.0	3.0	90.57	100	0.39	0.4	0.76
Spleen	2.0	2.0	93	100	0.2	0.15	0.32
**Plan 2**							
SABR_PTV	50.0	47.3	91.88	43.74	1.26	5.69	11.15
Duodenum	19.3	17.6	90	93.13	0.17	2.77	3.84
Stomach	2.9	2.6	95.44	100	0.31	0.27	1.62
Spinal cord	3.0	2.6	86	99.5	0.2	0.82	2.36
Spleen	2.5	2.2	92	100	0.37	0.11	1.09
**Patient B Plan 1**					
SABR_PTV	56.0	52.7	94.76	49.18	7	3	6.77
Duodenum	36.9	36.1	96.64	89.31	0.8	0.53	4.06
Stomach	12.6	12.6	97	100	0	0.1	2.41
Spinal cord	10.0	10.0	95.38	100	0.1	0.97	0.8
Spleen	5.1	5.1	96	100	0.2	0	0.93
**Plan 2**							
SABR_PTV	54.6	48.7	86.93	25.29	4.19	9.93	12.39
Duodenum	22.5	19.7	89.23	98.45	1	3	6.77
Stomach	15.0	12.9	89	89	0.3	1.53	6.45
Spinal cord	10.7	9.7	88.65	95.85	0.22	1.68	3.26
Spleen	4.5	3.9	92	100	0.29	0.59	1.99

For the duodenum adjacent to the stent, dose deviations reached up to 7.9%, with a GPR of 96%. Mean doses were 21.36 Gy (TPS) and 20.1 Gy (MC) (Table 3). Other OARs (stomach, spinal cord, and spleen) demonstrated high agreement and minimal differences.

For Plan 2 (OAR‐prioritized), TPS underestimated the lateral PTV dose by up to 13.1%, with a GPR of 43.74%. The mean PTV dose difference was 5.4%. For the duodenum, dose deviations reached 6.7%, with a GPR of 93.13% and mean dose difference of 8.92%.

**FIGURE 2:**
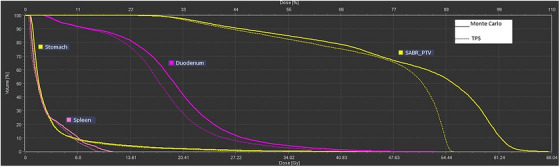
DVH of plan 2 for patient A.

**FIGURE 3:**
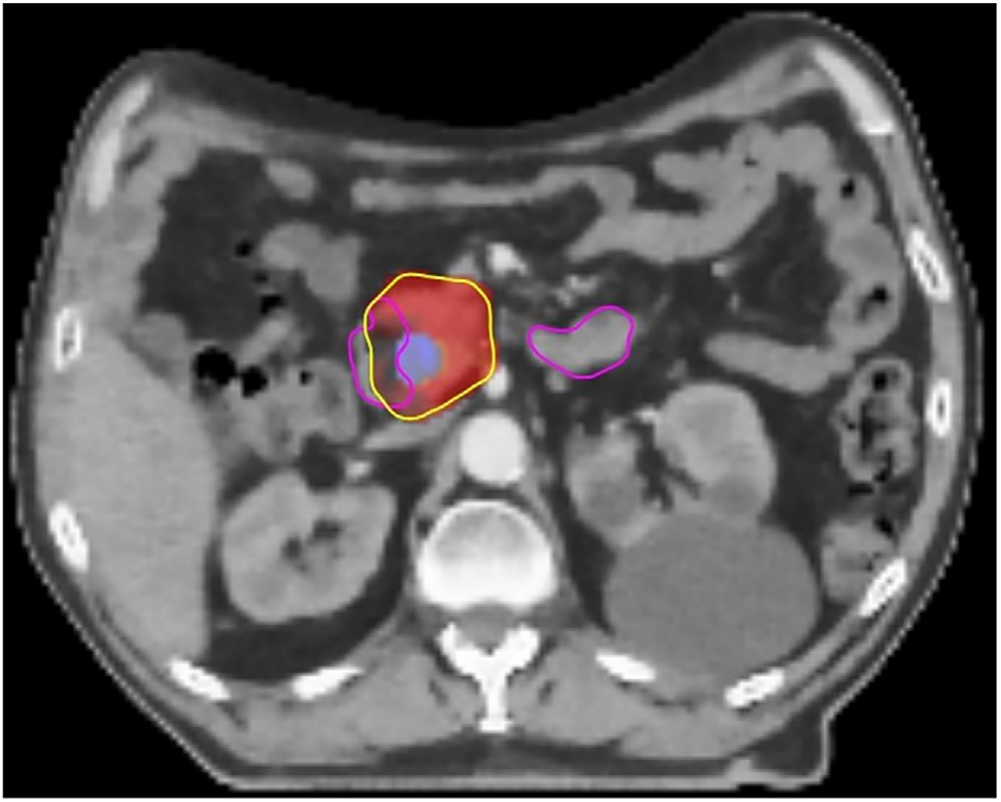
Spatial dose difference map (modified MC minus TPS) for Patient A, Plan 2, the same case shown in Figure 2. The mean PTV dose difference between models was 2.7 Gy. However, the perturbation is not uniformly distributed: red region indicate localized TPS underestimation reaching up to 8% of the prescription dose (approximately 4.0 Gy) lateral to the stent and near the duodenum, while blue regions indicate localized overestimation reaching 12.2% (approximately 6.1 Gy) in other regions.

MC: Monte Carlo; TPS: treatment planning system; PA: percentage agreement; GPR: gamma passing rate (3%/3 mm criteria); PTV: planning target volume; OAR: organ at risk; SABR_PTV: stereotactic ablative radiotherapy planning target volume; D95%, D50%, and D5%: dose received by 95%, 50%, and 5% of the structure volume, respectively.

Detailed numerical results for both plans are provided in Table 3, and mean dose values for Plan 2 are summarized in Table 3. DVH and spatial dose comparisons are shown in Figures 2 and 3; corresponding dose differences and gamma analysis maps for Patient A are provided in Figure S2.

For Patient B (Plan 1), TPS underestimated the dose by up to 11.2% in regions adjacent to the stent and overestimated it by 4.8% elsewhere. The PTV GPR was 49.18%, with 94.76% agreement. Mean PTV doses were 52 Gy (TPS) and 56 Gy (MC). For the duodenum near the stent, TPS underestimated the dose by 9.7% and overestimated it by 4%. Mean doses were 35.2 Gy (TPS) and 36.9 Gy (MC), with 96.64% agreement and an 89.31% GPR. Other OARs demonstrated high agreement between simulations.

For Plan 2 (OAR‐prioritized), TPS underestimated the lateral PTV dose by up to 12.3%, with reduced GPRs. The mean PTV dose difference was 10.8%. For the duodenum, dose deviations reached 7.7%, with a mean dose difference of 12.52%. Other OARs demonstrated smaller differences.

**FIGURE 4:**
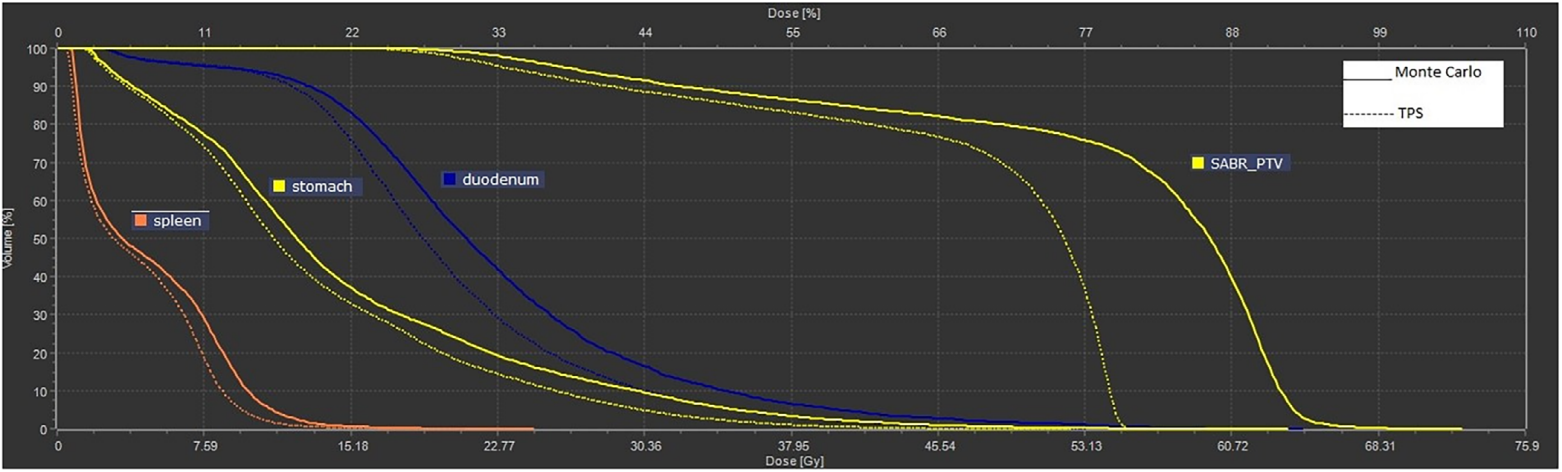
DVH of a plan 2 for patient B.

**FIGURE 5:**
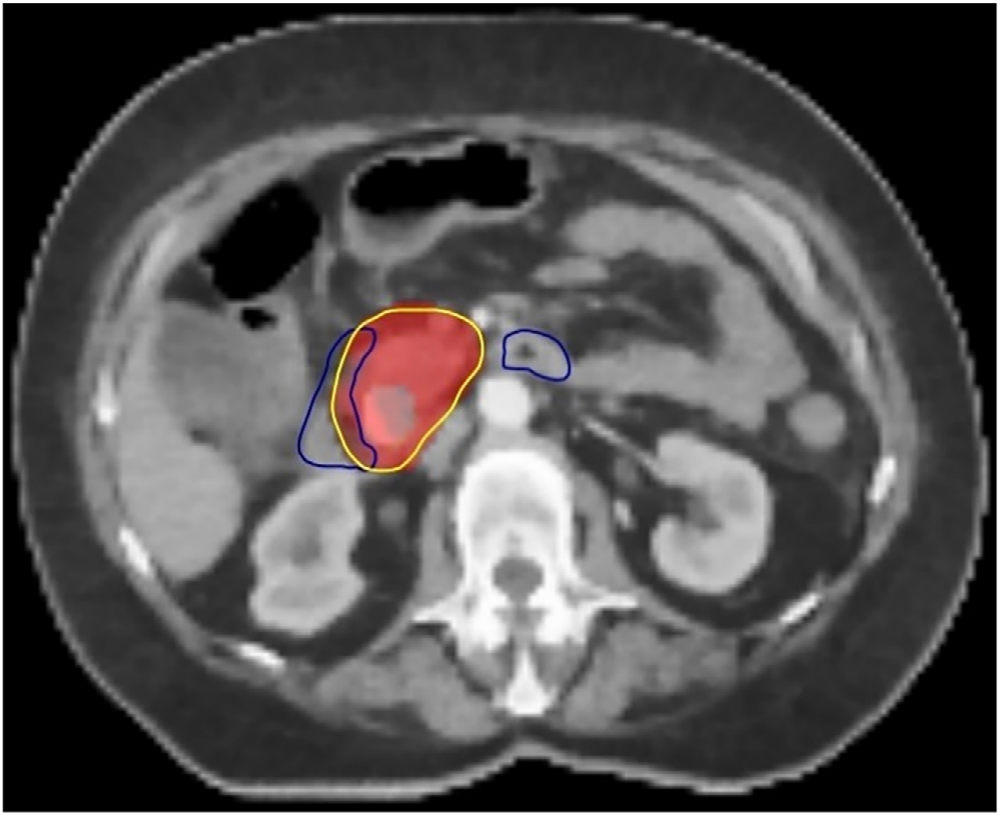
Spatial dose difference map (modified MC minus TPS) for Patient B, Plan 2, the same case shown in Figure 4. The mean PTV dose difference between models was 5.9 Gy. localized TPS underestimation reached 11.2% (approximately 5.6 Gy) adjacent to the stent, while overestimation reached 4.8% (approximately 2.4 Gy) elsewhere.

Comprehensive numerical comparisons are presented in Table 3. Mean doses for Plan 2 are summarized in Table 3. DVH and spatial comparisons are shown in Figures 4 and 5; The VMAT plan, dose difference and gamma analysis maps for Patient B are provided in Figure S3.

## 4 DISCUSSIONS

The effect of biliary stents on dose distribution is increasingly relevant as adaptive radiotherapy workflows are adopted across both MR‐guided and CBCT guided platforms. Online and offline adaptive replanning relies on accurate dose computation in the presence of patient specific anatomical features, including implants (16). The methodology proposed in this study, separation of the artefact affected inner lumen from the metallic mesh, followed by material reassignment within Monte Carlo dose calculation framework is platform uncertain and applicable wherever stent induced dose perturbation must be modelled accurately. It may be particularly impactful in the MR linac setting because the on table TPS algorithms MR‐RT systems currently do not incorporate a full Monte Carlo engine; However, the same correction principles apply to dose verification and second check calculations on conventional linacs.^33^, ^34^

Upon reviewing the original contouring, the stent appeared to be delineated as a solid structure. However, inspection of the CT images revealed a substantial difference between the HU values of the stent wall and its internal region. The stent is hollow and designed to expand against the wall to prevent blockage. The internal HU values were approximately ‐500, which is inconsistent with bile fluid and therefore unlikely to represent the true internal composition. This discrepancy is attributed to beam hardening, a well‐known CT artifact. When an X‐ray beam passes through high‐density material, lower‐energy photons are preferentially absorbed, increasing the mean photon energy of the beam—a phenomenon known as beam hardening.^35^, ^36^ This can produce dark streaks in high‐density areas such as bones and metal. Beam hardening can cause reductions in HU values within or adjacent to dense materials, such as inside the stent, commonly referred to as the cupping effect artifact. Although increasing tube voltage may reduce beam hardening, it compromises tissue contrast and increases patient radiation exposure, which is undesirable. Metal artifacts are particularly problematic in nitinol stents due to the high atomic numbers of nickel and titanium.^37^ Identifying these artifacts is essential for improving dose calculation accuracy. Contouring the entire stent region without correcting for internal HU distortion results in an overestimation of stent volume and an inaccurate representation of material composition.^38^, ^39^ In this study, refinement of the contouring approach reduced the modeled stent volumes from 26.1 to 22.8 cm^3^ (Patient A) and from 19.87 to 18.23 cm^3^ (Patient B), providing a more realistic representation. Failure to correct HU distortions may artificially increase or decrease calculated dose due to incorrect material assignment.

The use of the MC algorithm in conjunction with the new contouring method enhanced the accuracy of dose calculations. Lee et al. reported proximal dose increases between 2.3%‐8.2% using a single‐beam setup, with a minor increase of 1.8%.^26^ However, that study employed only one beam and did not reflect clinical treatment techniques for pancreatic cancer. Single‐beam simulations are useful for exploring fundamental dose perturbations but do not represent clinical VMAT delivery. VMAT has been shown to reduce distal dose enhancement compared with single‐beam arrangements.^26^ In the present study, distal dose enhancement was reduced but remained clinically relevant.

The localized dose enhancement at the stent edge is a manifestation of the well characterized high‐Z/tissue interface dose perturbation pattern. At megavoltage photon energies, Compton scattered electrons produced within nitinol are forward peaked but include a non‐negligible back scatter component, depositing additional dose immediately upstream of the proximal metal tissue interface (the backscatter dose perturbation factor per AAPM TG‐63).^39^ Distal to the metal attenuation of primary photons reduces dose, partially offset by forward scattered electrons in the first few millimeters beyond the exist interface. The net pattern proximal enhancement and distal reduction is consistent with prior high Z implant studies.^38^, ^40^ This pattern is captured by the modified MC model because the metal lumen geometry and the alloy composition are both correctly represented; it is substantially smeared in the standard model because the uncorrected CT HU assignment treated the stent interior as bone equivalent material, broadening the apparent metal extent and washing out the interface signature.

The proposed correction has been applied to the same patients under three beam configurations; a single anterior beam, VMAT with conventional fractionation (50.4 Gy in 28 fractions), and VMAT with SABR fractionation (50 Gy in 5 fractions). Under single beam irradiation, dose enhancement at the stent tissue interface reached 1.8% (Patient A) and 1.6% (Patient B), and distal dose reduction reached 4.9% and 4.8% respectively (modified vs standard MC) substantially larger than the 2% enhancement observed under VMAT. The modified MC vs TPS comparison under singe beam geometry showed proximal underestimation of 4% and distal overestimation of 6.2% in Patient A. The progressive attenuation of the scatter related enhancement from single beam (1.8%) through VMAT conventional (1.1%) to VMAT SABR (2%) is consistent with multi directional beam averaging and with Lee et al. phantom findings.^26^ The within patient design of this comparison eliminates inter patient anatomical variability as a confounding factor and confirms that the correction is technique independent. Because the correction operates at the level of CT to material assignment and Monte Carlo transport rather than beam optimization, application to 3D‐CRT, fixed field IMRT or VMAT SABR would follow the identical workflow. A direct head‐to‐head comparison of VMAT, fixed field IMRT and 3D CRT plans for the same SABR patients remains a target for future work.

Direct geometric modelling of the stent mesh for example, by generating an STL representation of the stent geometry and importing it into the MC simulation environment was considered as an alternative methodology. The contouring material correction approach was chosen for three reasons. First, the TPS dose grid voxel size (2.5 × 2.5 × 3.0 mm^3^) is an order of magnitude larger than typical nitinol stent dimension (150‐200 µm), so vowelizing a stent level STL onto the dose grid collapses back to an effective shell representation and dose not yield a meaningful improvement in geometric reliability at the resolution at which dose is computed. Second, the proposed workflow operates entirely within the standard DICOM/TPS/MC pipeline, the three RT structures were created in Mirada Medical and transferred directly to PRIMO without requiring specialist CAD software or sub voxel geometry handling. Third, the approach is design agnostic at the workflow level: the inner lumen mesh separation generalizes across stent geometries whereas an STL workflow would require manufacturer specific re modelling of the stent pattern for each stent design. A direct comparison between as STL based reference model and the present vowelized approach including investigation of sub millimeter dose perturbations at the individual stent level is identified as valuable future work for benchmarking purposes. The methodological core of the correction, separation of the artefact affected inner region from the metallic mesh followed by material reassignment is independent of stent design. Therefore, the proposed workflow should generalize to silicon covered stents, braided nitinol meshes and double stent configurations. The magnitude of dose perturbation, however, is design dependent: Lee et al. reported up to 19.5% enhancement for double bare metal stents under single beam geometry, versus 2.7% to 8.2% for single bare metal stents.^26^ Recharacterization for each stent design therefore remains necessary.

Unlike phantom‐based investigations, this study incorporated patient DICOM datasets and SABR planning constraints, which influence beam modulation and OAR sparing. Compared with single beam phantom studies, the present simulation framework, patient DICOM data with clinical SABR constraints and VMAT delivery better reflects the conditions of contemporary pancreatic SABR planning. Prospective multi patient evaluation and phantom based experimental verification remain necessary, however, before quantitative clinical generalisations can be made. A dose‐escalation approach was applied in this work. The MC algorithm can accurately model dose build‐up and backscatter effects from high‐atomic‐number materials, whereas conventional TPS algorithms may not fully account for these interactions. Importantly, MC simulations alone were insufficient when CT artifacts and material representation were not corrected, as shown by the discrepancies observed between the standard and modified MC models. Using the modified model reduced uncertainty in dose estimation by correcting both CT artifacts and material composition. This improvement may allow safer dose escalation to the target while maintaining OAR protection. Clinically, a low GPR within the PTV is unacceptable in a region that is otherwise homogeneous, where the primary heterogeneity arises from the stent. With emerging trends toward dose escalation in pancreatic cancer to improve biological response, accurate dose modeling becomes increasingly important. Significant disagreement between planned and delivered dose distributions may compromise treatment effectiveness and safety.

The AAPM TG‐218 report recommends 3%/2 mm as the contemporary IMRT/VMAT QA action limit, with tighter criteria appropriate for SABR treatments.^32^ In the present study, PTV GPR values at 3%/3 mm, the most lenient clinical criterion were already well below the 95% universal tolerance recommended by TG‐218, reaching as low as 25.29% (Patient B, Plan 2). Since tighter gamma criteria (for example 2%/2 mm, 1%/1 mm) impose strictly more stringent passing conditions, GPR values at these thresholds can only be equal to or lower than those at 3%/3 mm. The magnitude of the observed failures at 3%/3 mm therefore establishes that the modified MC vs TPS discrepancy exceeds clinically acceptable thresholds regardless of criterion choice, reinforcing the conclusion that conventional TPS algorithms exhibit clinically significant inaccuracies in the presence of metallic biliary stents.

The Plan 1 (PTV prioritized) vs Plan 2 (OAR prioritized) comparisons reveal a clinical informative asymmetry. In Plan 1, the optimizer pushes dose toward the PTV with relatively relaxed OAR sparing, producing larger absolute PTV dose discrepancies between TPS and modified MC (PTV D50% difference of 5.2 Gy in patient A and 3.3 Gy in Patient B). In Plan 2, the optimizer shapes dose around the OARs, narrowing the PTV dose inconsistency but emphasizing the relative OAR discrepancy, particularly in the duodenum, where the dose gradient passes immediately adjacent to the stent. This asymmetry implies that the clinical magnitude of stent induced TPS error is plan strategy dependent.

The absolute dose values underscore the clinical relevance of the observed discrepancies. For Patient A (Plan 1), TPS reported a mean PTV dose of 54 Gy, whereas the modified MC model yielded 47.2 Gy, a difference of 6.8 Gy that places the delivered PTV dose below the prescribed 50 Gy. On the contrary, for Patient B (Plan 1), the modified MC model indicated a mean PTV dose of 56 Gy compared with 52.7 Gy reported by TPS, suggesting PTV received 3.3 Gy more than the planning system predicted. The opposing direction of these discrepancies between patients demonstrates that stent induced dose perturbation is not a systematic bias open to a uniform correction factor; rather, it is patient specific and plan specific, dependent on stent position relative to the PTV (Figure S‐1), beam trajectory distribution, and optimizer weighting. For the duodenum, the structure most at risk in pancreatic SABR, the modified MC model consistently indicated higher doses than TPS: in Patient B (Plan 2), the mean duodenal dose as 22.5 Gy compared with 19.7 Gy reported by TPS, an underestimation of 2.8 Gy. Given that the duodenal constraint for this protocol is D0.5cc < 33 Gy (optimal) to 35 Gy (mandatory), an unrecognized dose increase of this magnitude could contribute to patients exceeding their tolerance threshold without clinical detection. Structure distant from the stent (stomach, spinal cord, spleen) demonstrated mean dose difference of less than 0.5 Gy between models, confirming that the perturbation is localized to the stent region and does not spread to distant organs at risk.

Translating the observed dosimetric discrepancies into biological effective dose (BED, *α*/*β* = 10 Gy) clarifies their potential clinical significance. The 50 Gy in 5 fraction prescription corresponds to a planned BED 10 of 100 Gy. A 5% under delivery (the lower end of the range observed in mean PTV dose) corresponds to a delivered BED 10 of approximately 93 Gy; 10% under delivery corresponds to approximately 83 Gy. Published dose response analyses indicate that response rates in pancreatic adenocarcinoma approach 50% only at BED 10 to 100 Gy^7^, and that dose escalation above 70 Gy BED is associated with improved survival.^9^, ^41^ The discrepancies observed therefore lie within a range that could possibly affect local control if TPS reported dose systematically overestimates delivered dose. A causal link to clinical outcomes cannot, however, be inferred from two patient dosimetric series and must be examined in prospective studies with adequate power.

In addition to the aforementioned limitations, this study only included data from 2 patients, which limited the amount of statistical information available, thereby limiting the ability to generalize the findings to a greater number of patients. It was also noted that similar dose discrepancy amounts occurred in both case studies; however, it would take a larger group of patients to assess whether the discrepancies occur similarly in varying anatomical configurations and different locations within the body where the stent is placed.

Further, only one type of biliary stent was analyzed (nitinol alloy Cook Evolution). As biliary stents vary widely in design, as well as manufacturing materials, wall thickness, mesh density, and many other parameters, it cannot be assumed that the same degree of dose perturbation occurs in all types of biliary stents, and future research involving a variety of biliary stent types would be needed to confirm the findings of this study.

The analytical anisotropic algorithm was the only treatment planning system utilized for this study's comparisons. Newer TPS algorithms, such as the Acuros XB algorithm, are capable of calculating radiation doses through highly dense, heterogeneous media more accurately than the analytical anisotropic algorithm. Future studies utilizing Monte Carlo simulations and comparing them to newer TPS algorithms will help identify whether TPS‐related discrepancies exist when using newer dose calculation engines.

Although the modified contouring method improves upon treating the stent as a homogenous high‐density object, the manual delineation step introduces an observer dependent component of uncertainty. Three factors influence this (i) the CT acquisition protocol (tube voltage, slice thickness, and whether a metal artefact reduction reconstruction kernel has been applied, the present study used a Philips Big bore scanner at 120 kVp with the IMR1 iterative reconstruction kernel, which partially mitigates metal artefacts compared to standard filtered back projection). (ii) operator familiarity with the appearance of high Z‐artefacts on planning CT, and (iii) inter observer variability in identifying the metal lumen boundary slice by slice. To place this uncertainty in quantitative context ± 1 mm perturbation of the inner‐lumen boundary (approximately one CT pixel: 1.17 mm) would affect a thin shell of voxels at the mesh‐lumen cross section (from the artefactual ‐500 HU to the corrected 1000 HU). The dose differences between the standard and modified MC models (14.9‐15.7% PTV overestimation) substantially exceed any plausible contouring boundary uncertainty, confirming that the methodological gain from material correction dominates the residual uncertainty introduced by manual delineation. A formal ± 1 mm sensitivity analysis with MC re‐simulation is identified as future work to quantify the residual contouring uncertainty precisely. in routine clinical use, this step would also benefit from semi‐automated lumen segmentation tools. Development and validation of such a toll is identified as future work.

The most important methodological limitation of this study is the absence of an experimental measurement of dose in the presence of a biliary stent. A future phantom verification employing (i) radiochromic film at the stent tissue interface to capture in plane dose gradients, (ii) thermoluminescent or typically stimulated luminescent dosimeters at controlled distance proximal and distal to the stent to verify the back scatter/ forward perturbation pattern predicted by the modified MC, and (iii) cross validation against a fully measured VMAT delivery within an anthropomorphic abdominal phantom containing an actual SEMS, would provide the experimental ground truth that the present simulation only framework lacks. Such a study is identified as the next stage of this investigation.

Intra‐ and inter‐fraction movement of the pancreas was not taken into consideration in this analysis. Movement of the pancreas due to breathing and digestion can cause changes in the spatial relationships between the stent, target volume, and surrounding organs at risk; thus potentially altering both the magnitude and/or locations of dose perturbations. Studies examining motion management and/or utilizing four‐dimensional computed tomography data would provide a more complete understanding of dose perturbations caused by biliary stents.

Lastly, the potential clinical consequences of the observed dose discrepancies in terms of local tumor control and treatment toxicities were not evaluated. Future prospective clinical studies evaluating dose discrepancies with clinical endpoints would be required to evaluate clinical importance and determine appropriate dose escalation protocols in the presence of biliary stents.

## 5 CONCLUSION

The modified model provides an alternative approach to improve the dose distribution accuracy in pancreatic cancer patients with biliary stents, which is particularly important for dose escalation. The model incorporates accurate stent material composition and a refined contouring technique. Two comparisons were performed: the first between the standard MC model and the modified MC model, and the second between the modified MC model and the TPS. The results demonstrated that the modified MC model produced meaningful differences in PA and GPR, as well as in dose distribution within the target and surrounding OARs. MC simulation alone did not improve agreement with TPS unless material properties were updated and CT beam hardening effects were addressed.

Although the VMAT technique reduced dose enhancement, distal dose perturbations remained present. The modified model reduces treatment uncertainty in patients with pancreatic stents by improving dose calculation accuracy and enabling more reliable dose escalation strategies. As uncertainty decreases, estimation of radiotherapy‐related side effects may also improve. Given the evidence supporting dose escalation for improved outcomes in pancreatic cancer, enhancing dose calculation accuracy is essential to minimize unintended dose to OARs while maintaining effective target coverage.

## AUTHOR CONTRIBUTION

A.A.: Conceptualization, Methodology, Data curation, Formal analysis, Investigation, Writing original draft, O.N.: Conceptualization, Writing—Review & Editing, R.P.H.: Conceptualization, Writing—Review & Editing

## FUNDING INFORMATION

The authors received no financial support for the research, authorship and publication of this article.

## CONFLICT OF INTEREST STATEMENT

The author have nothing to report.

## Supporting information



Supporting Infomation

Supporting Infomation

Supporting Infomation

## Data Availability

The data that support the findings of this study are available from the corresponding author upon reasonable request.
