# Supplementary figures and images for: Assessment of dose distribution in pancreatic SABR in the presence of a metallic biliary stent using Monte Carlo modeling
Source: J Appl Clin Med Phys. 2026 Jul 22;27(8):e70712. doi: 10.1002/acm2.70712 (PMC13389806; doi:10.1002/acm2.70712)

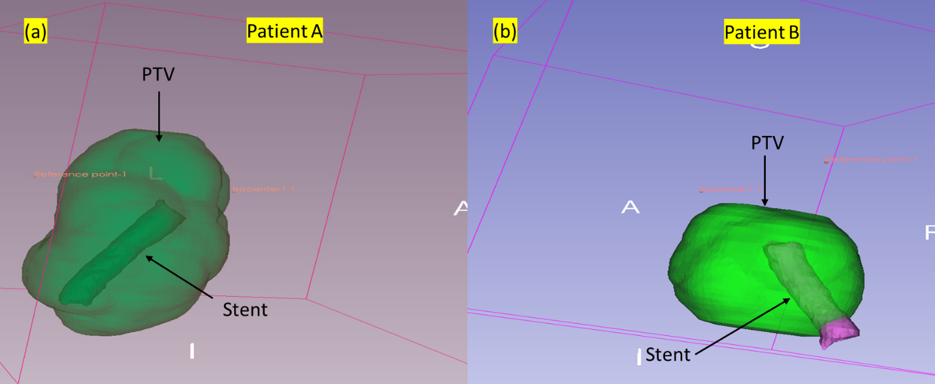

Supplement: Supplementary file 1 — Supporting Infomation [file ACM2-27-e70712-s003.tif]

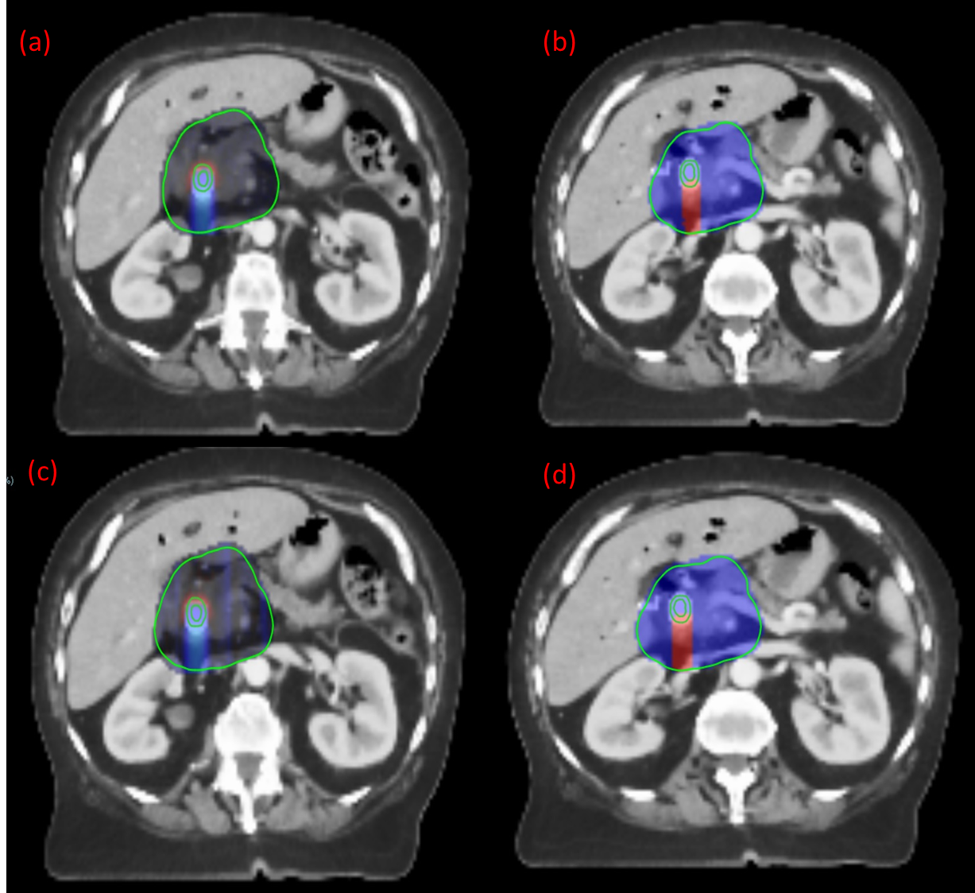

Supplement: Supplementary file 2 — Supporting Infomation [file ACM2-27-e70712-s002.tif]

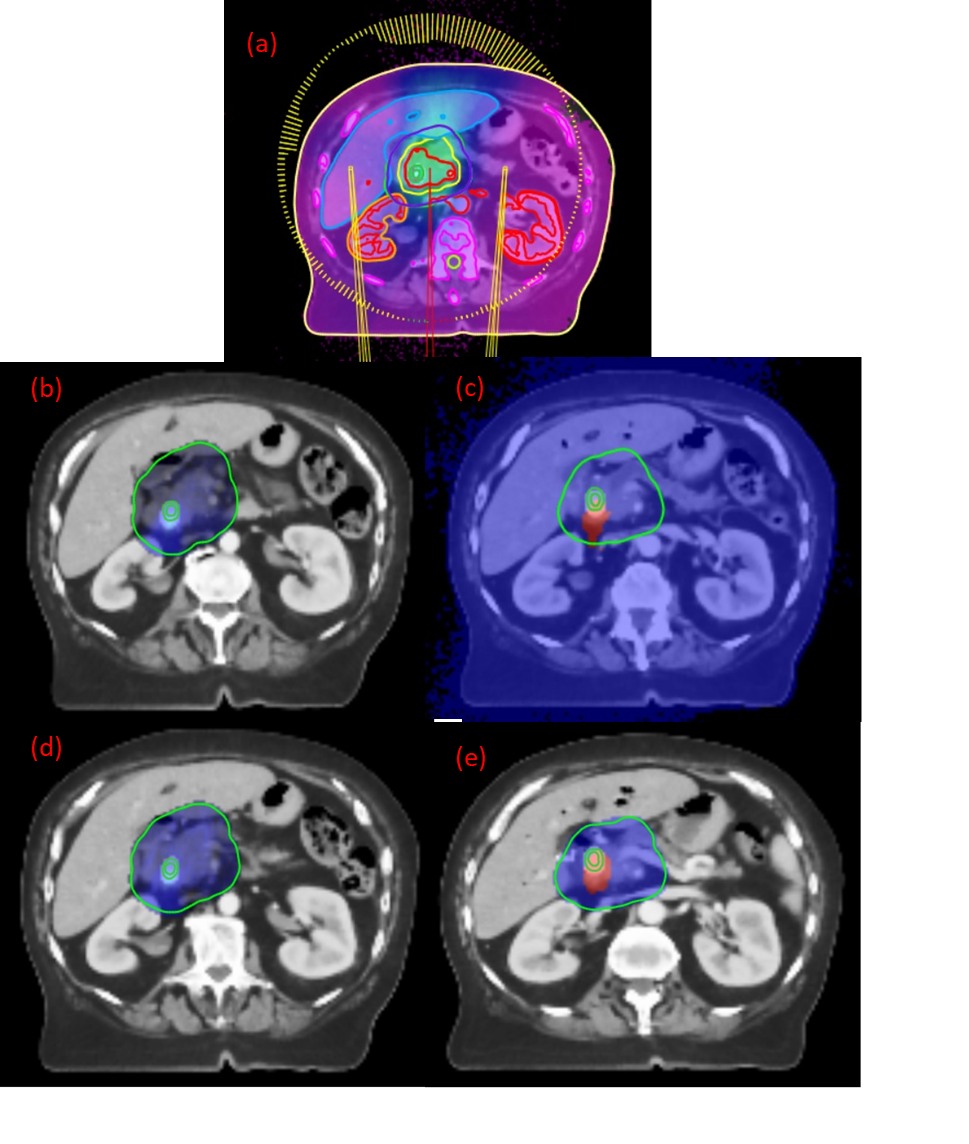

Supplement: Supplementary file 3 — Supporting Infomation [file ACM2-27-e70712-s001.tif]
